# Impaired microvascular circulation in distant organs following renal ischemia

**DOI:** 10.1371/journal.pone.0286543

**Published:** 2023-06-02

**Authors:** Jesus H. Dominguez, Danhui Xie, K. J. Kelly

**Affiliations:** 1 Department of Medicine, Division of Nephrology, Indiana University School of Medicine, Indianapolis, Indiana, United States of America; 2 Department of Medicine, Renal Section, Roudebush Veterans’ Affairs Medical Center, Indianapolis, Indiana, Unites States of America; Uniformed Services University, UNITED STATES

## Abstract

Mortality in acute kidney injury (AKI) patients remains very high, although very important advances in understanding the pathophysiology and in diagnosis and supportive care have been made. Most commonly, adverse outcomes are related to extra-renal organ dysfunction and failure. We and others have documented inflammation in remote organs as well as microvascular dysfunction in the kidney after renal ischemia. We hypothesized that abnormal microvascular flow in AKI extends to distant organs. To test this hypothesis, we employed intravital multiphoton fluorescence imaging in a well-characterized rat model of renal ischemia/reperfusion. Marked abnormalities in microvascular flow were seen in every organ evaluated, with decreases up to 46% observed 48 hours postischemia (as compared to sham surgery, p = 0.002). Decreased microvascular plasma flow was found in areas of erythrocyte aggregation and leukocyte adherence to endothelia. Intravital microscopy allowed the characterization of the erythrocyte formations as rouleaux that flowed as one-dimensional aggregates. Observed microvascular abnormalities were associated with significantly elevated fibrinogen levels. Plasma flow within capillaries as well as microthrombi, but not adherent leukocytes, were significantly improved by treatment with the platelet aggregation inhibitor dipyridamole. These microvascular defects may, in part, explain known distant organ dysfunction associated with renal ischemia. The results of these studies are relevant to human acute kidney injury.

## Introduction

Acute kidney injury (AKI) is a major clinical challenge [1–3]. AKI occurs very frequently in critically ill patients with ~1.7 million deaths annually [4–6]. An enlarging body of data supports the systemic harmful effects of AKI [3, 7–11]. Mortality in AKI is most often due to dysfunction of extra-renal organs [5, 12, 13] and not renal failure, per se; mortality increases with the number of failed organs [14]. That may be one reason for the lack of specific, effective treatments for AKI; multiple potential treatments have been shown to provide marked protection from AKI in animals [15], yet none have been translated to human therapy. Mortality is greater in patients with AKI than those with end stage kidney disease, consistent with the detrimental systemic effects of AKI [16]. Inflammatory mediators in the brain [17] and impaired locomotor function, have been found in murine AKI [18]. Infiltration of neutrophils, macrophages and T-lymphocytes, cytokine induction and cell death are found in the small intestine in experimental AKI [19, 20]. In the liver, hepatocyte apoptosis, increased alanine aminotransferase [21, 22] and tumor necrosis factor expression [20, 23] have been found after renal ischemia. AKI is also associated with adverse effects on the cardiac and pulmonary systems [24–27]. Remote organ dysfunction was felt to be a factor in the lack of efficacy in the synthetic atrial natriuretic peptide [28] and insulin-like growth factor [29] trials in human AKI. Nevertheless, the pathophysiology of distant organ effects of renal injury is currently not well understood. Understanding the linkage of renal injury with remote organ effects is critical in developing effective treatments for preventing and reducing morbidity and mortality in AKI [11, 16, 29]. The purpose of the present study was an investigation of microvascular abnormalities in extra-renal organs after renal ischemia and reperfusion. We hypothesized that the microvascular coagulation and impaired perfusion seen in the kidney postischemia [30] would also impact remote organs.

## Materials and methods

### Please see supplemental material for detailed methods

#### Animal Protocols

The protocol was approved by the Institutional Animal Use and Care Committee (Roudebush VA and Indiana University School of Medicine). After ensuring adequate anesthesia, male Sprague-Dawley rats were subjected to 20 minutes bilateral renal ischemia with non-traumatic microaneurysm clamps (85 grams of pressure, Roboz, Gaithersburg, MD) as described [31–38]. Sham surgery consisted of an identical surgical procedure with the exception of application of microaneurysm clamps. In some experiments, dipyridamole (2, 2’,2”,2”‘-(4,8-di(piperidin-1-yl)pyrimido[5,4-*d*]pyrimidine-2,6-diyl)bis (azane-triyl) tetraethanol) or vehicle (25% ethanol) was administered intraperitoneally at the time of renal ischemia or 24 hours later. There was no difference between sham surgery/vehicle and sham surgery/dipyridamole in the measured parameters and thus only the sham surgery/vehicle values are presented. Systolic blood pressure, serum creatinine, red blood cell (RBC) aggregation on peripheral smears and fibrinogen were quantified. Six animals per group were randomly assigned.

#### Intravital multi-photon fluorescence microscopy [30, 32, 34]

Microvascular flow was quantified 24 and 48 hours post-ischemia by intravital imaging using an Olympus Fluoview 1000 confocal/multiphoton microscope, Olympus Corporation, Center Valley PA. Briefly, after ensuring adequate anesthesia with thiobarbital (30mg/kg), organs to be imaged were exteriorized and Hoechst (500ug in 0.5ml physiological saline, Molecular Probes, Eugene, OR) was injected intravenously to allow visualization of nuclei. Microvascular plasma flow was visualized after infusion of fluorescein conjugated albumin (Molecular Probes, 2mg in 0.5ml physiological saline) and serial images acquired approximately every 0.5 second. Plasma flow was quantified as movement of dye-excluding erythrocytes in line scans (with correction for microvascular angle) [30]. We focused on the longer (48 hour) time point when serum creatinine had improved to near baseline levels, but preliminary studies (S1 Fig in S1 File) did show similar results at the 24 hour time point. Rouleaux were identified as shadows of “stacked coins” moving together in blinded, serial intravital images. Intravascular leukocytes were identified as nucleated (staining with Hoechst) cells within the vasculature. The white blood cells (WBC) were classified as adherent if not free flowing or rolling but stationary (on the capillary wall) for at least 10 seconds. All quantification was performed without knowledge of experimental group. Additional measures of inflammation included myeloperoxidase activity [38, 41] and polymerase chain reaction for complement component C3 and intercellular adhesion molecule-1 (ICAM) [30] measured in tissue.

**Statistics.** Data are expressed as means ± 1 standard error. Analysis of variance was used to determine if differences among mean values reached statistical significance. Tukey’s test was used to correct for multiple comparisons. Fishers’ exact test was used for categorical data. The null hypothesis was rejected at p<0.05. Statistics and data analysis were performed in a blinded fashion, without knowledge of experimental group.

## Results

### Baseline parameters and the effect of renal ischemia

**Fig 1:**
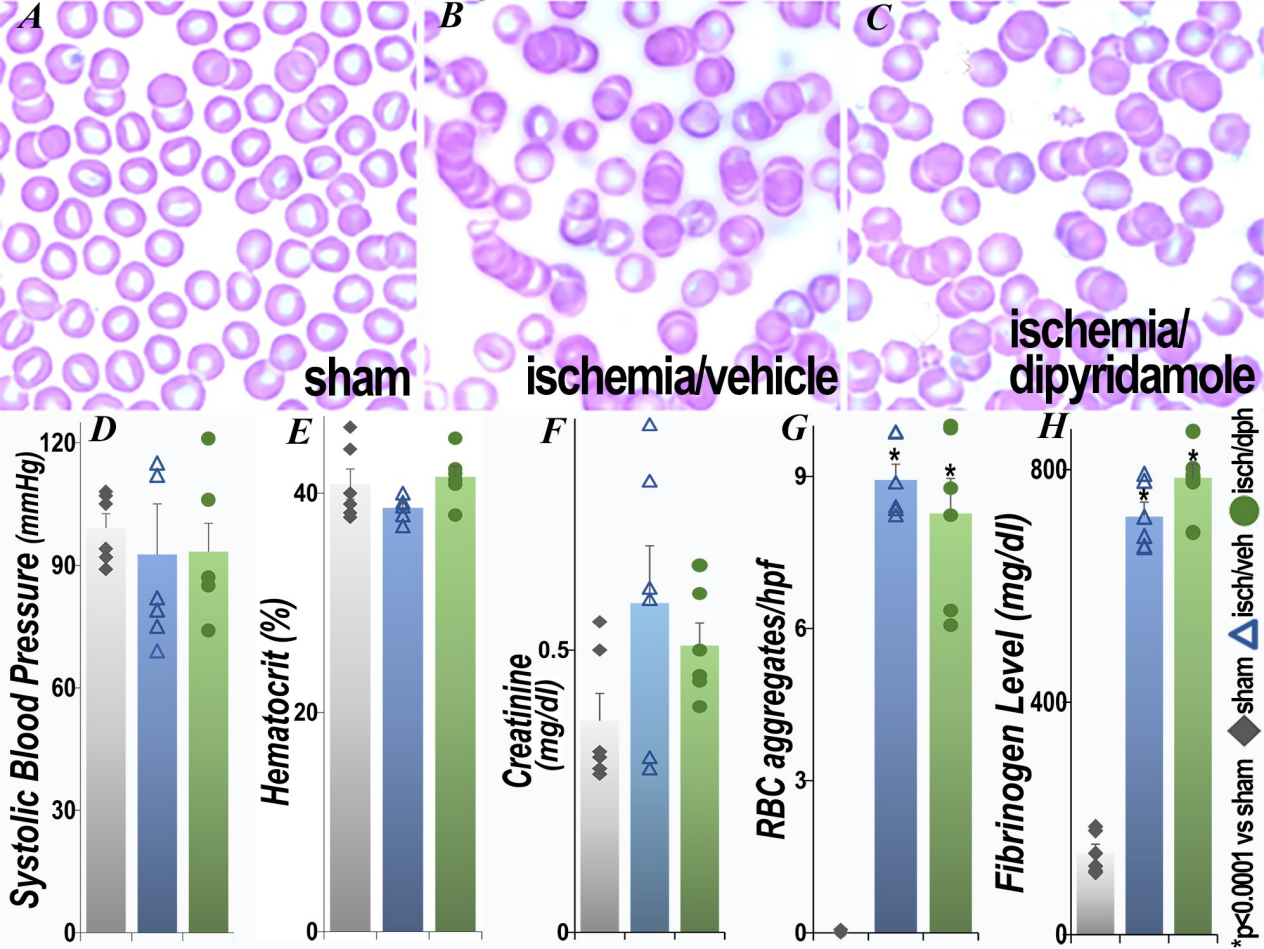
Effect of renal ischemia on peripheral blood erythrocyte aggregation. Representative Wright’s stained peripheral smears (A-C) and quantification of systolic blood pressure (D), hematocrit (E), serum creatinine (F), erythrocyte aggregates(G) and fibrinogen levels (H) 48 hours after surgery are presented. There was no difference between sham surgery/vehicle and sham surgery/dipyridamole in the measured parameters and thus only the sham surgery/vehicle values are presented in this and subsequent figures. Each group contained 6 animals. In this and subsequent figures, mean values ± 1 standard error of the mean are presented. Statistical analysis employed ANOVA. *p<0.0001 vs sham (exact p values are in **S1 File**); isch, ischemia; veh, vehicle; dph, dipyridamole.

Systolic blood pressure was no different among all groups, sham surgery and postischemia. Similarly, there was no difference in hematocrit among the sham and postischemia groups (Fig 1). Mean serum creatinine increased 24 hours after ischemia in both the ischemia/vehicle and ischemia/dipyridamole groups, to 0.76 ± 0.14 and 0.55 ±0.05 mg/dl, respectively (p<0.05 vs sham), but was not significantly different between the two postischemia groups (p = 0.187). By 48 hours, there were no statistically significant differences in mean serum creatinine among all groups. Erythrocyte aggregation [39, 40] and abnormal RBC morphology (Fig 1) were observed in the peripheral blood after renal ischemia and was not improved by dipyridamole. Serum fibrinogen was significantly elevated after ischemia (vs sham) and was similar in the vehicle and dipyridamole postischemia groups (p = 0.13). Thus, the two postischemia groups were comparable.

### Effect of renal ischemia on vascular flow in extra renal organs

Significantly impaired microvascular blood flow was observed in live images of all remote organs examined 48 hours after renal ischemia, when serum creatinine was no different from sham levels in all groups. Decreased plasma flow postischemia was accompanied by microvascular thrombi and evidence of inflammation, including leukocytes adherent to microvascular endothelium. Tissue myeloperoxidase (MPO) activity and complement component C3 and intercellular adhesion molecule-1 (ICAM) by PCR were measured as indices of tissue inflammation.

### Effect of renal ischemia on the brain

**Fig 2:**
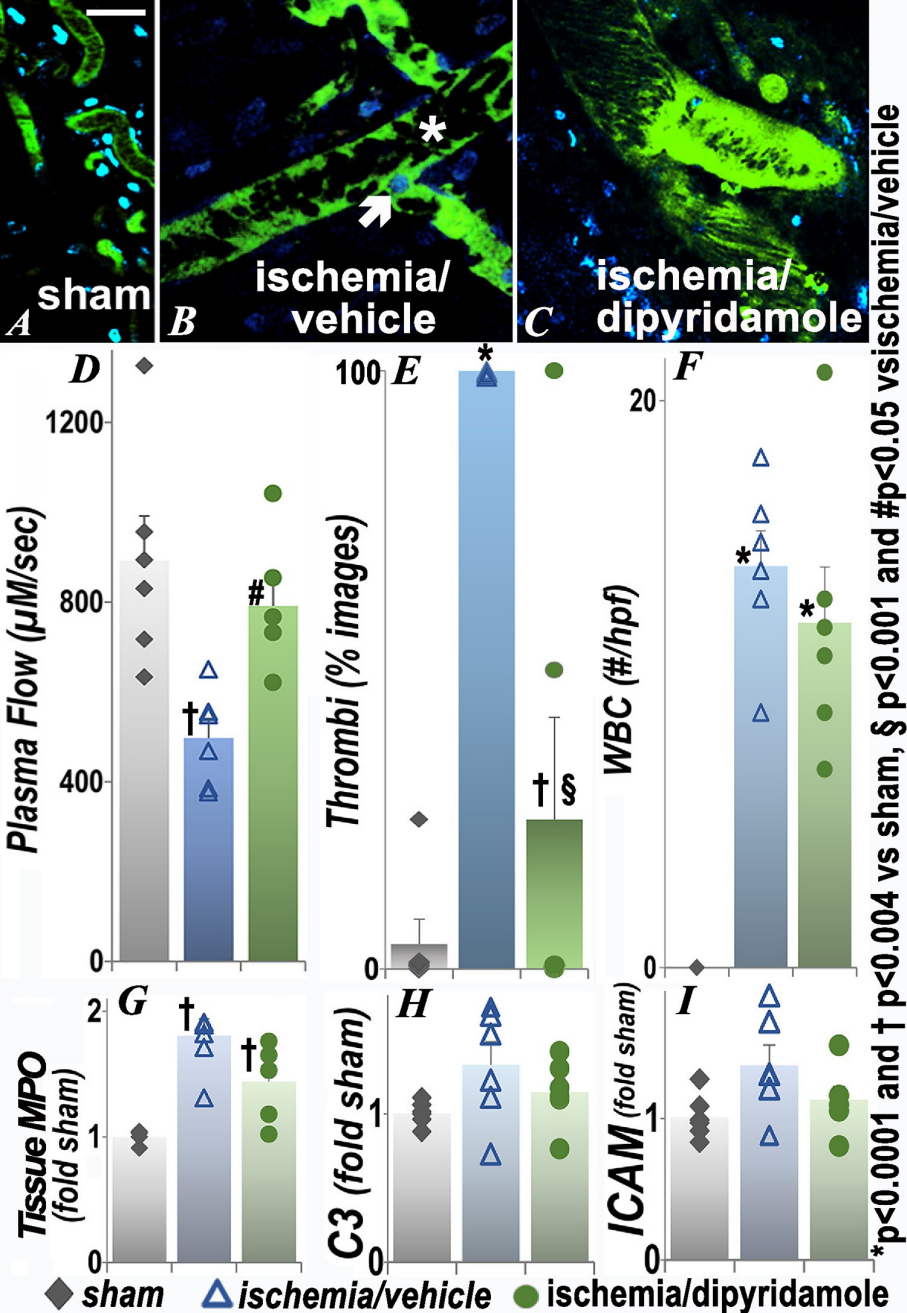
Effect of renal ischemia on leukocyte retention and vascular flow in the brain. Representative images of brain 48h after renal ischemia or sham surgery and quantification of plasma flow, thrombi, adherent WBC, tissue MPO activity and C3 and ICAM transcript levels are presented. In this and subsequent figures, the vascular space is delineated with fluorescein-conjugated albumin (green) and the nuclei labeled with Hoechst (blue). The arrow shows an example of a nucleated cell (WBC) adherent to the vessel in live images. During intravital imaging, obstruction then reversal of flow was seen at the asterisk. Each group contained 6 animals. For thrombi, percent of total fields are presented (numerator and denominator values for this and subsequent figures are in S1 File). In this and subsequent figures, statistical analysis for plasma flow, number of WBC, MPO, C3 and ICAM employed ANOVA; for categorical data (thrombi), Fisher’s exact test was used. *p<0.0001 and †p<0.004 vs sham; §p<0.001 and #p<0.05 vs ischemia/ vehicle (exact p values are in S1 File); scale bar is 100 μm.

In the brain (Fig 2), significantly impaired microvascular flow was accompanied by reversal of flow in some areas (Fig 2B, asterixis). Quantification of microvascular flow as the velocity of dye-excluding erythrocytes showed significantly decreased blood flow in the brain (Fig 2D) postischemia to approximately 50% of the sham values. Microvascular plasma flow in the ischemia/dipyridamole group was significantly higher than the ischemia/vehicle group (p = 0.048), so that flow was restored to levels that were no different than those in the sham group. In addition to impaired microvascular flow, erythrocyte aggregation and leukocytes (some clearly multilobed, consistent with neutrophils) adherent to vascular endothelial cells were observed. Stacked RBC (rouleaux, microthrombi) were seen in all images acquired after renal ischemia, but only 1 of 24 obtained after sham surgery (Fig 2E). The number of rouleaux was significantly attenuated in the group that received dipyridamole at the time of renal ischemia. In intravital images, leukocytes (nucleated intravascular cells) adherent to the vessel wall were also observed as an apparent cause of impaired microvascular flow. The number of adherent WBC was significantly higher 48 hours after ischemia than after sham surgery (Fig 2F), but the effect of dipyridamole was primarily on clotting. The effects at 24 hours (S1 Fig in S1 File) were similar; dipyridamole did not result in significant improvements when given after ischemia (S2 Fig in S1 File). Tissue myeloperoxidase activity (MPO), an additional measure of inflammation, was also significantly increased in the brain postischemia (Fig 2G). The decrease with dipyridamole did not reach statistical significance (p = 0.052). There was no statistical difference among groups in tissue complement component C3 or intercellular adhesion molecule-1 (ICAM), perhaps because the inflammation was largely peri-vascular. There were no differences in extravascular histology between sham surgery and postischemia groups (S3 Fig in S1 File).

### Effect of renal ischemia on the small intestine

**Fig 3:**
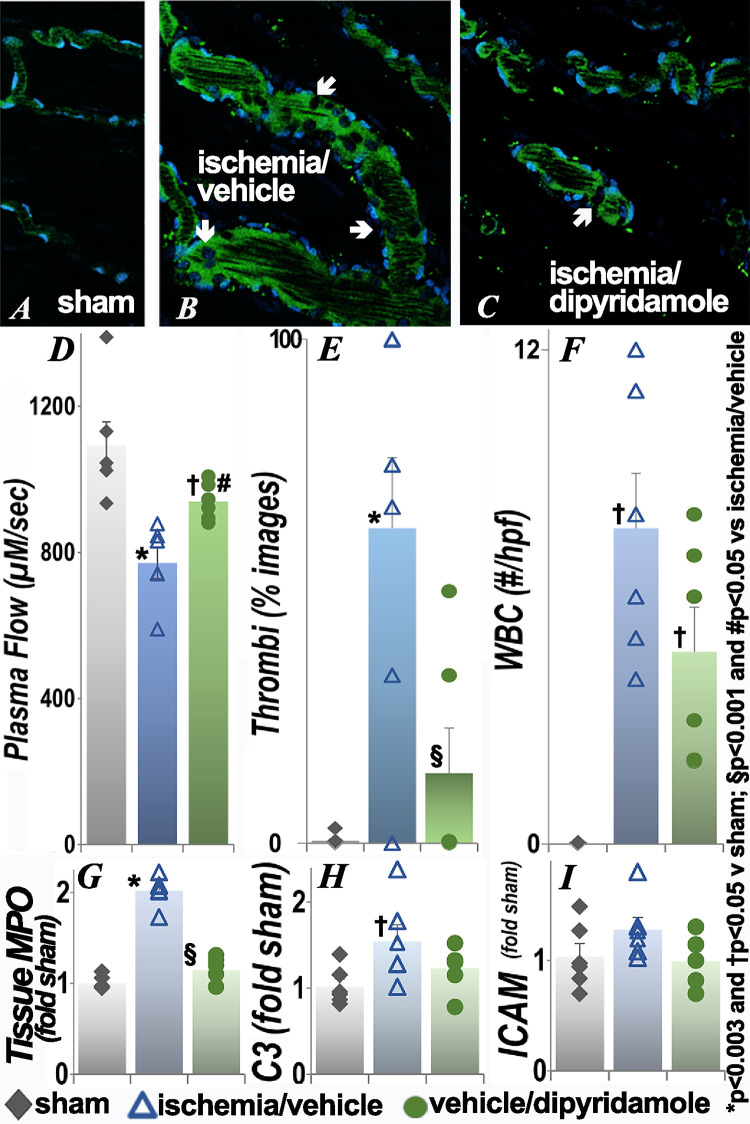
Effect of renal ischemia on leukocyte retention and vascular flow in the small intestine. Representative images of intestinal microcirculation 48h after renal ischemia or sham surgery and quantification of plasma flow, microthrombi, adherent WBC, tissue MPO activity and C3 and ICAM transcript levels are presented. The arrows show adherent nucleated leukocytes in live images. Each group contained 6 animals. Statistical analysis for plasma flow, number of WBC, MPO, C3 and ICAM employed ANOVA; for categorical data (thrombi), Fisher’s exact test was used. *p<0.003 and †p<0.05 vs sham; §p<0.001 and #p<0.05 vs ischemia/vehicle (exact p values are in **S1 File**); scale bar is 100 μm.

Significantly impaired microvascular flow with microthrombi and adherent WBC were also observed in the intestine (Fig 3) with improvement in plasma flow and thrombi seen in the ischemia/dipyridamole group (Fig 3D and 3E). Vascular dilation and decreased plasma flow were seen proximal to obstructing WBC in live images (Fig 3B). The changes in plasma flow and thrombi were also seen at 24 hours (S1 Fig in S1 File); dipyridamole did not result in significant improvements when given after ischemia (S2 Fig in S1 File). Tissue myeloperoxidase activity (Fig 3G) and transcript levels of complement component C3 (Fig 3H), but not ICAM (Fig 3I), were also significantly increased in the intestine following renal ischemia. Tissue myeloperoxidase was improved in the dipyridamole group. Extravascular histology was similar in the three groups (S3 Fig in S1 File).

### Effect of renal ischemia on the mesenteric circulation

**Fig 4:**
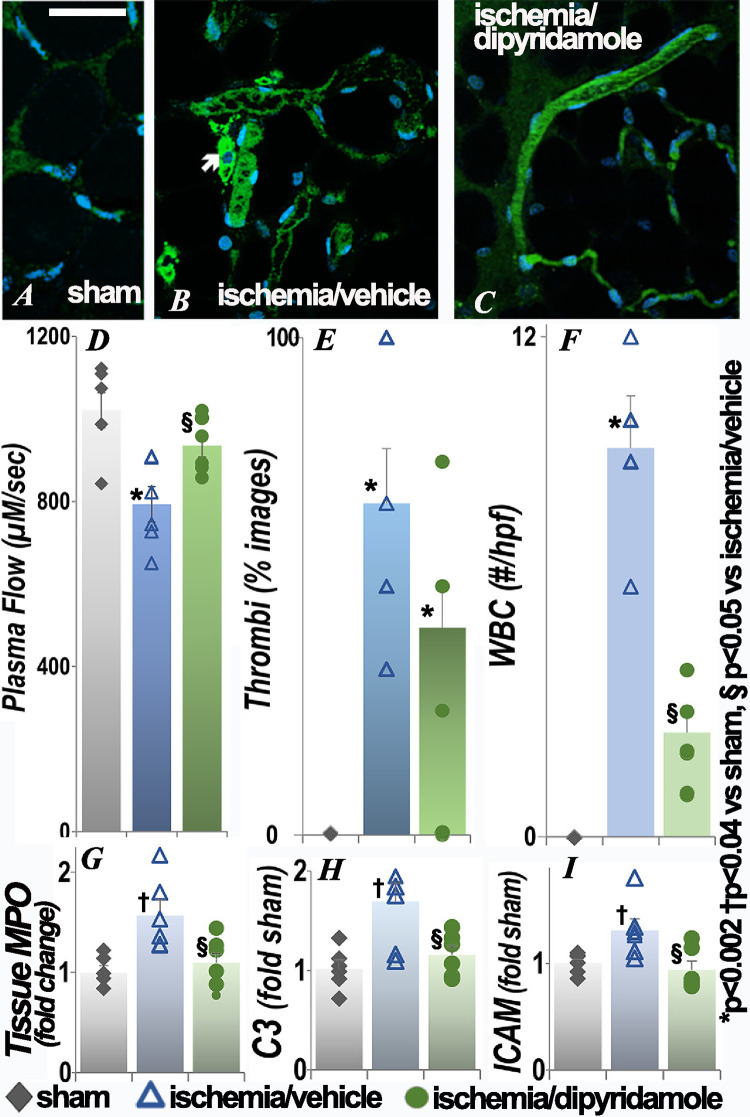
Effect of renal ischemia on leukocyte retention and vascular flow in the mesentery. Representative images of mesenteric microvasculature 48h after renal ischemia or sham surgery, quantification of plasma flow, rouleaux, adherent leukocytes, tissue MPO activity and C3 and ICAM transcript levels are presented. The arrow points to RBC aggregation and intravascular nuclei (WBC) adherent to vessels in live images. Each group contained 6 animals. Statistical analysis for plasma flow, number of WBC, MPO, C3 and ICAM employed ANOVA; for categorical data (thrombi), Fisher’s exact test was used. *p<0.002, † p<0.04, vs sham; §p<0.05 vs ischemia/vehicle (exact p values are in **S1 File**); scale bar is 100 μm.

Decreased microvascular flow, although to a lesser degree than in the brain or small intestine, was seen in the mesentery following renal ischemia (Fig 4). Thrombi in the microvasculature and adherent WBC were also observed in the mesentery, with retention of dye proximal to adherent WBC (Fig 4B). Microvascular flow was restored to sham surgery levels and intravascular WBC were significantly improved in the postischemia group treated with dipyridamole (Fig 4D and 4F). Alterations in plasma flow, thrombi and adherent were also seen at 24 hours (S1 Fig in S1 File); dipyridamole did not result in significant improvements when given after ischemia (S2 Fig in S1 File). Tissue myeloperoxidase activity (Fig 4G) and complement component C3 (Fig 4H) and ICAM (Fig 4I) transcript levels, were also significantly increased in the mesentery following renal ischemia and improved in the dipyridamole group. Extravascular histology was similar in the three groups (S3 Fig in S1 File).

### Effect of renal ischemia on the liver

**Fig 5:**
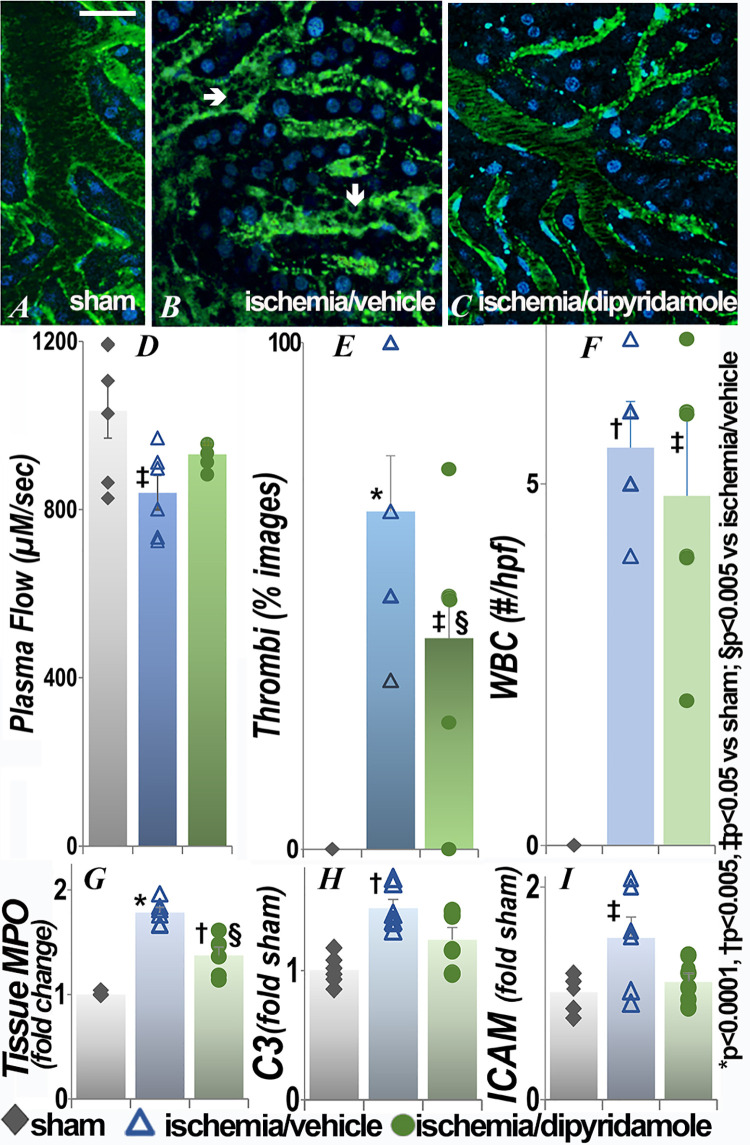
Effect of renal ischemia on leukocyte retention and vascular flow in the liver. Representative images of the hepatic microcirculation 48h after renal ischemia or sham surgery, quantification of microvascular plasma flow, erythrocyte aggregates, adherent WBC, tissue MPO activity and transcript levels of C3 and ICAM are presented. Examples of erythrocyte aggregation and intravascular leukocytes adherent to vessels, resulting in retention of the fluorescent dye, are at arrows. Each group contained 6 animals. Statistical analysis for plasma flow, number of WBC, MPO, C3 and ICAM employed ANOVA; for categorical data (thrombi), Fisher’s exact test was used. *p<0.0001, † p<0.005, ‡p<0.05 vs sham, §p<0.005 vs ischemia/vehicle (exact p values are in S1 File); scale bar is 100 μm.

Decreased in microvascular plasma flow along with microthrombi and adherent WBC were seen in the liver 48 hours after renal ischemia (Fig 5), with improvement in microthrombi in the dipyridamole group (Fig 5E). Improvement in microvascular flow with dipyridamole did not reach statistical significance in the liver (p = 0.08, Fig 5D). Alterations in plasma flow, thrombi and adherent WBC were also seen at 24 hours (S1 Fig in S1 File); dipyridamole did not result in significant improvements when given after ischemia (S2 Fig in S1 File). Tissue myeloperoxidase activity (Fig 4G) and complement component C3 (Fig 5H) and ICAM (Fig 5I) transcript levels were also significantly increased in the liver following renal ischemia. Tissue myeloperoxidase a measure of neutrophil content, was improved in the dipyridamole group. Extravascular histology was similar in the three groups (S3 Fig in S1 File).

### Effect of renal ischemia on the spleen

**Fig 6:**
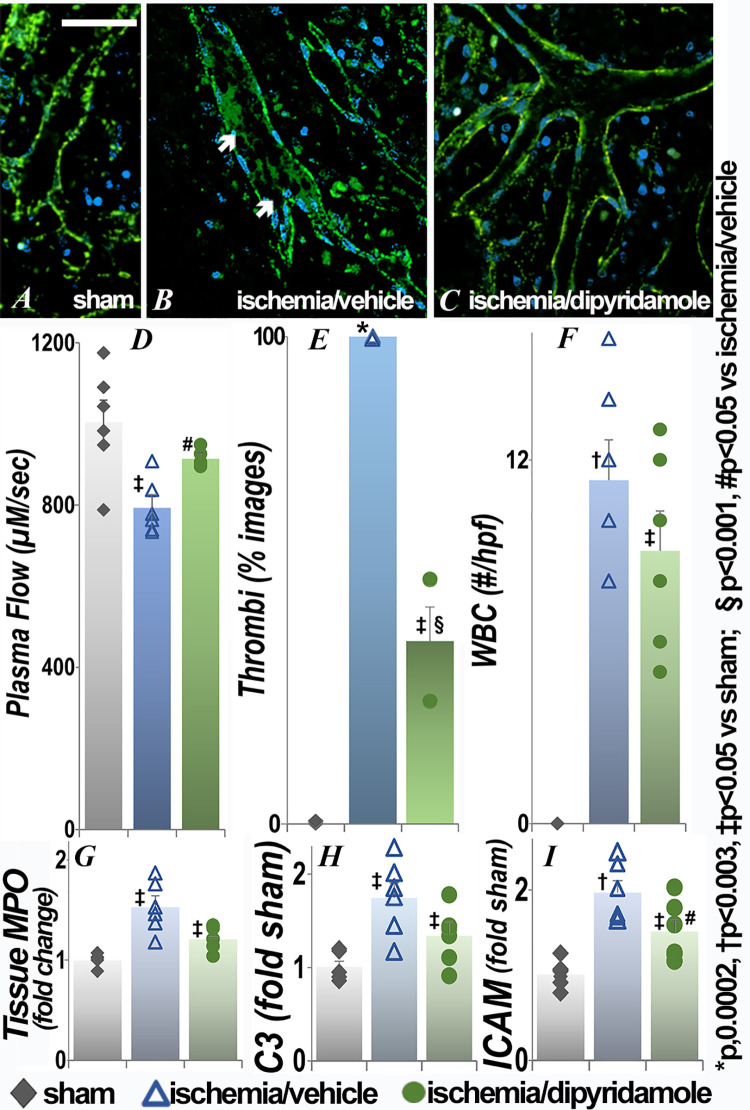
Effect of renal ischemia on leukocyte retention and vascular flow in the spleen. Representative images of the microvasculature of the spleen show areas of RBC aggregation and adherent leukocytes (examples at arrows) 48 hours after renal ischemia as compared to sham surgery. Quantification of microvascular flow, RBC aggregates, adherent leukocytes, tissue MPO activity and C3 and ICAM transcription after sham surgery or renal ischemia are also presented. Each group contained 6 animals. Statistical analysis for plasma flow, number of WBC, MPO, C3 and ICAM employed ANOVA; for categorical data (thrombi), Fisher’s exact test was used. *p<0.0002, † p<0.003, ‡p<0.05 vs sham; §p<0.002, #p<0.05 vs ischemia/vehicle (exact p values are in S1 File); scale bar is 100 μm.

Decreased in microvascular plasma flow along with microthrombi and adherent WBC were also seen in the spleen 48 hours after renal ischemia (Fig 6), with improvement in each of these parameters in the dipyridamole group. Alterations in plasma flow, thrombi and adherent were also seen at 24 hours (S1 Fig in S1 File). Dipyridamole did not result in significant improvements when given after ischemia (S2 Fig in S1 File). Tissue myeloperoxidase activity (Fig 6G) and complement component C3 (Fig 6H) and ICAM (Fig 6I) transcript levels, were also significantly increased in the spleen following renal ischemia. ICAM was improved in the dipyridamole group. Extravascular histology was similar in the three groups (S3 Fig in S1 File).

### Effect of ischemia on microvascular plasma flow, microthrombi and adherent leukocytes in the kidney

**Fig 7:**
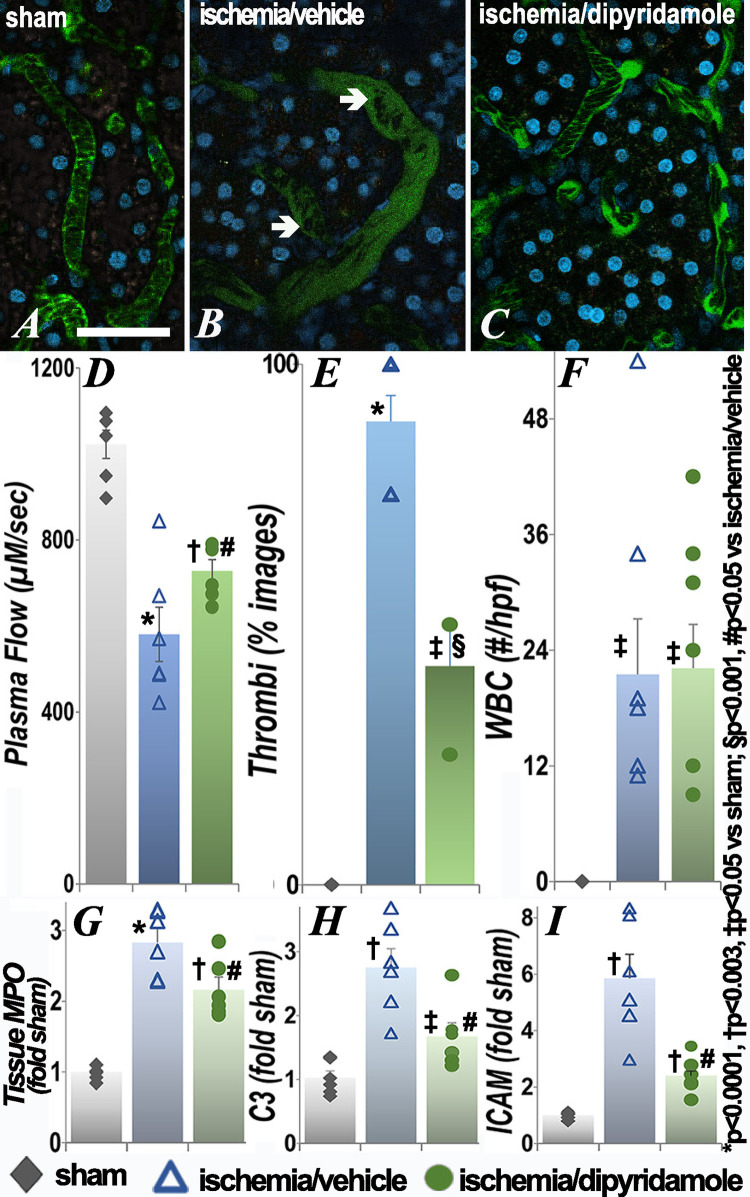
Effect of renal ischemia on leukocyte retention and vascular flow in the kidney. Representative images of the renal microvasculature show areas of RBC aggregation and adherent leukocytes (arrows) with retention of administered fluorophore 48 hours after renal ischemia as compared to sham surgery. The graphs show quantification of microvascular plasma flow, thrombi, adherent WBC, tissue MPO activity and C3 and ICAM transcript levels. Each group contained 6 animals. Statistical analysis for plasma flow, number of WBC, MPO, C3 and ICAM employed ANOVA; for categorical data (thrombi), Fisher’s exact test was used. *p<0.0001, † p<0.003, ‡p<0.05 vs sham; §p<0.001, #p<0.05 vs ischemia/vehicle (exact p values are in S1 File); scale bar is 100 μm.

Decreases in microvascular plasma flow along with microthrombi and adherent WBC were prominent in the kidney, the organ subjected to ischemia, and persisted after reperfusion (Fig 7). Microvascular flow and thrombi were improved after treatment with dipyridamole. Alterations in plasma flow, thrombi and adherent were also seen at 24 hours (S1 Fig in S1 File). Dipyridamole did not result in significant improvements when given after ischemia (S2 Fig in S1 File). Tissue myeloperoxidase activity (Fig 6G) and complement component C3 (Fig 6H) and ICAM (Fig 6I) transcript levels, were also significantly increased following renal ischemia and improved in the dipyridamole group. Extravascular histology demonstrated tubular injury and casts postischemia (S3 Fig in S1 File).

### Effect of ischemia on the heart

**Fig 8:**
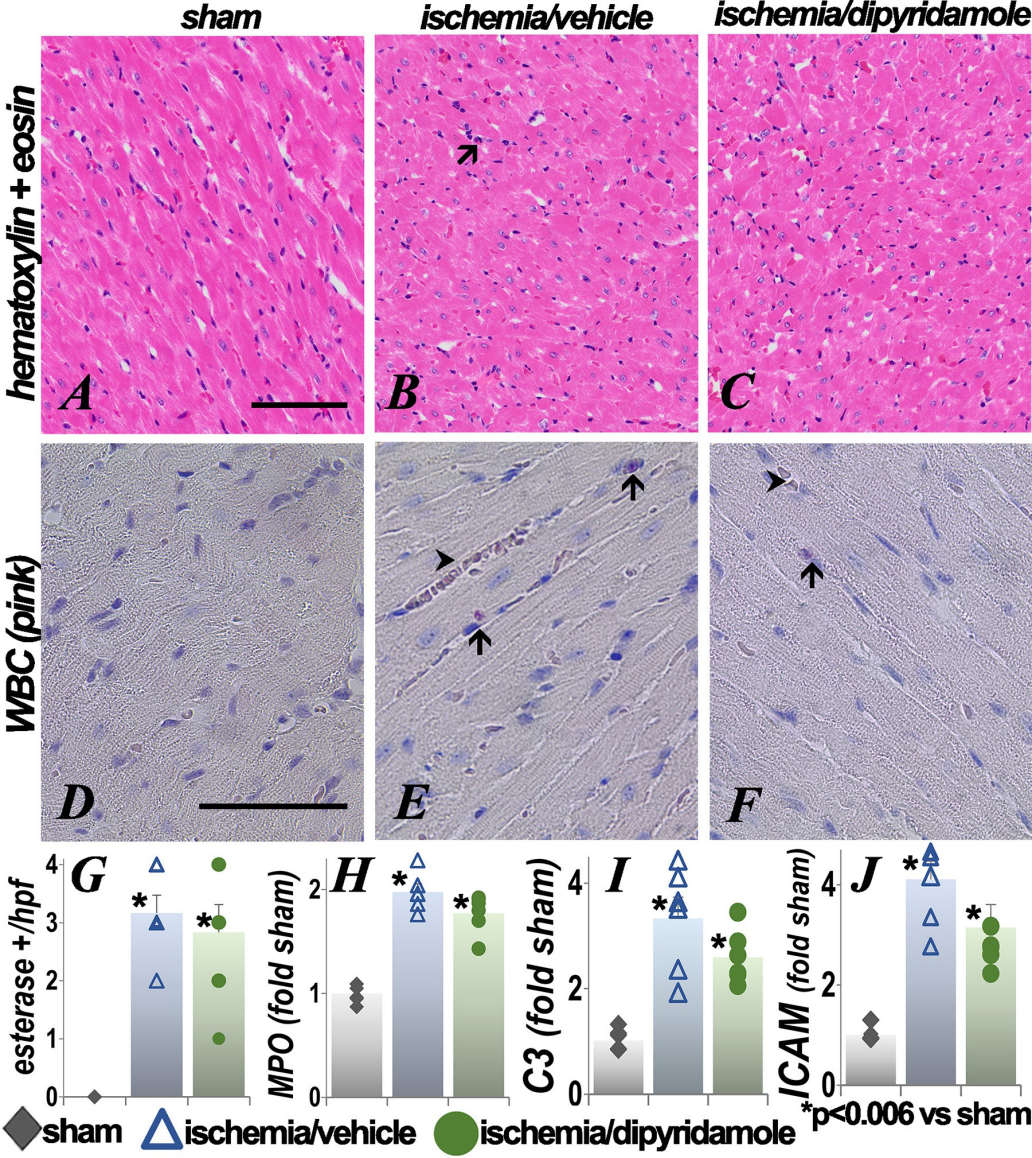
Effect of renal ischemia on the heart. Representative images of the hematoxylin and eosin and esterase stained heart sections 48 hours after renal ischemia or sham surgery are presented. Arrows show leukocytes and arrowhead RBC aggregates. The graphs show quantification of tissue MPO activity and C3 and ICAM transcript levels. Each group contained 6 animals. Statistical analysis employed ANOVA. *p<0.006 vs sham (exact p values are in S1 File); scale bar is 100 μm.

Cardiac abnormalities have been demonstrated following renal ischemia [41, 42], yet the heart is not amenable to intravital microscopy to assess plasma flow as it would require cardioplegia and thus not be physiological. In hematoxylin and eosin stained sections (Fig 8B), rare leukocytes were seen in the postischemia hearts. Esterase staining showed increased neutrophils in the heart after renal ischemia (Fig 8D–8G). Tissue myeloperoxidase activity and C3 and ICAM levels were also increased in the heart after renal ischemia (Fig 8H–8J). The inflammatory parameters were not improved in the dipyridamole group.

These results support the hypothesis that microvascular effects of renal ischemia extend to extra-renal organs, resulting in microvascular thrombi and decreased plasma flow. The effect of dipyridamole, an anti-platelet agent was primarily on clotting.

## Discussion

Acute kidney injury (AKI) occurs very frequently, particularly in critically ill patients and carries a high mortality rate [4, 5]. Although tremendous advances have been made in defining and diagnosing AKI, revealing the pathophysiology and in general care, the only FDA approved treatment remains supportive renal replacement therapy. An enlarging body of data indicates that distant organ effects of AKI are clinically important and contribute to morbidity and mortality [43]. Thus, understanding remote organ effects of AKI is necessary to improve AKI outcomes [11, 16, 29].

In the present study, we tested the hypothesis that the microvascular abnormalities and clotting seen in the kidney postischemia would also be present in remote organs. Significantly impaired microvascular flow with microthrombi was found in all organs evaluated after AKI in living animals. Importantly, these abnormalities occurred well after reperfusion, at a time when serum creatinine in the postischemia groups was not significantly different than in the sham animals. This indicates that the defects were due to ischemia rather than uremia, consistent with findings in patients where mortality in AKI is greater than that in end stage kidney disease [16]. Many factors can alter microvascular flow, including inflammation, erythrocyte aggregation and impaired deformability as well as hematocrit. We [33, 41, 44, 45] and others [46–51] have documented inflammation following renal ischemic injury. Although we did observe decreases in microvascular flow in proximity to obstructing leukocytes, our data indicate that inflammation was not the major factor in decreased microvascular plasma velocity in remote organs following renal ischemia in this model, since significant improvements in microvascular flow, but not adherent leukocytes, were observed with the platelet inhibitor dipyridamole. Greater changes in intravascular WBC than in whole tissue measures of inflammation (MPO, C3, ICAM) are consistent with remote organ inflammation being largely within the vasculature. In addition to directly obstructing blood flow, activated polymorphonuclear cells can also result in platelet aggregation and coagulation which impair blood flow [52–54]. RBC aggregation directly decreases plasma flow and can decrease oxygen transfer to tissues and thus exacerbate ischemia, creating a cycle of injury [55, 56]. We also found erythrocyte aggregates in the peripheral circulation following renal ischemia. RBC aggregation can negatively affect the endothelium, for example via suppression of nitric oxide synthesis [57]. We have demonstrated sustained renal microvascular clotting postischemia [30]. In the present study, we found impaired perfusion resulted in decreased fluorescence in the kidney 2 days after reperfusion, which may have implications for potential therapies reaching injured renal tissue. In addition, there was impaired microvascular plasma flow with microvascular thrombi in each remote organ examined following renal ischemia, with clotting resulting in reversal of flow in some areas of microthrombi formation. Microvascular blood flow and clotting were improved in the dipyridamole treated postischemia group. Dipyridamole, an inhibitor of platelet phosphodiesterase, was used to assess the role of platelets, rather than as a therapeutic agent. The lack of efficacy when given after reperfusion is consistent with the results showing that clotting was already present by 24 hours of reperfusion.

The increases in fibrinogen observed in this study can contribute to RBC aggregation. Fibrinogen is the major plasma protein coagulation factor. It is also an acute phase reactant [58], increased by inflammatory mediators, such as interleukin-6 [6, 59], known to rise after ischemia [30, 60]. Fibrinogen adheres to the surface of erythrocytes, form bridges between neighboring cells and thus facilitates aggregation [61]. It has been shown that increased RBC aggregation with increases in fibrinogen results in increased margination of leukocytes and increases in WBC-endothelial adhesion [62]. Fibrinogen and fragments of fibrinogen also increase inflammatory mediators, leukocyte adhesion and diapedesis, thus amplifying the effect on erythrocyte aggregation [63]. The increases in fibrinogen levels after renal ischemia found in this study likely contribute to rouleaux formation. Dipyridamole treatment was associated with decreased RBC aggregation, consistent with platelet-induced microthrombi contributing to the decreased plasma flow seen postischemia [64]. Increases in plasma viscosity can also decrease microvascular flow [65], although there was a lack of change in hematocrit in this study, so changes in viscosity were not likely significant. In transgenic sickle mice, a clinically relevant model of RBC aggregation [66], renal and extra-renal injury and mortality are more severe following renal ischemia [67]. Other factors known to cause RBC aggregation occur following renal ischemia, including hypoxia and alterations in pH.

In summary, remote organ inflammation is a recognized consequence of renal ischemia. This study shows other distant organ alterations—widespread, heterogeneous microvascular abnormalities—occur after renal ischemia. These abnormalities include impaired microvascular plasma flow with microthrombi formation in the brain, mesentery, liver, spleen and small intestine. The changes were amenable to therapy. If similar abnormalities occur in human AKI, alterations in microvascular flow in distant organs may be critical in the very high mortality seen clinically in AKI.

## Supporting information

S1 File (PDF)
