# Supplementary material for: Impaired microvascular circulation in distant organs following renal ischemia
Source: PLoS One. 2023 Jun 2;18(6):e0286543. doi: 10.1371/journal.pone.0286543 (PMC10237479; doi:10.1371/journal.pone.0286543)
Supplement: S1 File — (PDF) [file pone.0286543.s001.pdf]

SUPPLEMENTAL FIGURE LEGENDS

**FIG. S1.** *Effect of Renal Ischemia on the Microvascular Circulation in Remote Organs 24 hours after Renal Ischemia or Sham Surgery*. Quantification of plasma flow, microthrombi and adherent WBC in the brain, small intestine, mesentery, liver, spleen and kidney 24 hours after surgery are presented. The three groups are sham surgery, renal ischemia/vehicle and renal ischemia/dipyridamole (given at the time of ischemia). Each group contained 3 animals in this preparatory work. Statistical analysis for plasma flow and number of WBC employed ANOVA; for categorical data (thrombi), Fisher's exact test was used. *$p<0.008$, † $p<0.04$ vs sham; ‡$p<0.05$ vs ischemia/vehicle (exact p values are in supplemental table); scale bar, 100 µm

**FIG. S2.** *Effect of Renal Ischemia on the Microvascular Circulation in Remote Organs after Later Dipyridamole*. Quantification of plasma flow, microthrombi and adherent WBC in the brain, small intestine, mesentery, liver, spleen and kidney 48 hours after surgery are presented. The three groups are sham surgery, renal ischemia/vehicle and renal ischemia/dipyridamole (given 24 hours postischemia). Each group contained 3 animals in this preparatory work. Statistical analysis for plasma flow and number of WBC employed ANOVA; for categorical data (thrombi), Fisher's exact test was used. *$p<0.008$, † $p<0.04$ vs sham; ‡$p<0.05$ vs ischemia/vehicle (exact p values are in supplemental table); scale bar, 100 µm

**FIG. S3.** *Effect of Renal Ischemia on Extravascular Histology*. Representative images of brain, small intestine, mesentery, liver, spleen and kidney 48 hours after sham surgery or renal ischemia (with and without dipyridamole) are presented. Arrow shows tubular cell injury (blebbing) and arrowhead points to an intratubular cast. The scale bar, 100 µm

MATERIALS and METHODS

*Animal Protocols.* All experiments were conducted in strict accordance with the recommendations in "Guiding Principles for Research Involving Animals and Human Beings" and ARRIVE guidelines. The protocol was approved by the Institutional Animal Use and Care Committee (Roudebush VA and Indiana University School of Medicine). Male Sprague-Dawley rats (180-240g; Harlan Laboratories, Indianapolis, IN) were anesthetized with intraperitoneal (i.p.) pentobarbital (50 mg/kg) and placed on a homeothermic

table to maintain core body temperature at ~37$^{O}$C. After ensuring adequate anesthesia, both renal pedicles were occluded for 20 minutes with 85 gram pressure non-traumatic microaneurysm clamps (Roboz Surgical, Gaithersburg, MD) as described (30, 31, 35-38). Sham surgery consisted of an identical surgical procedure with the exception of application of microaneurysm clamps. In some experiments, dipyridamole (2, 2',2",2'"-(4,8-di(piperidin-1-yl)pyrimido[5,4-*d*]pyrimidine-2,6-diyl)bis(azanetriyl) tetra-ethanol, Sigma, St. Louis, MO, 10 mg/kg) or vehicle (25% ethanol) was administered IP at the time of renal ischemia or 24 hours following surgery. There was no difference between sham surgery/vehicle and sham surgery/dipyridamole in the measured parameters and thus only the sham surgery/vehicle values are presented. Blood was obtained via tail vein or cardiac puncture for determination of hematocrit as well as creatinine (by standard enzymatic reaction). Heparinized venous blood collected 48 hours after surgery was collected and stained in accord with the procedures of the International Council for Standardization in Haematology(39-40) and 20 random images per smear/animal were obtained and coded for the presence or absence of red blood cell (RBC) aggregates by a blinded observer. Fibrinogen was measured via ELISA according to the distributor's instructions (Aniara, West Chester, OH). Systolic blood pressure was measured by tail cuff using a IITC (Kent Scientific, Torrington, CT) amplifier prior to imaging [2]. Tissues fixed in paraformaldehyde were cut at 4um and stained with standard hematoxylin for histology and esterase [3] to identify polymorphonuclear neutrophils (in coded sections evaluated by a blinded observer). There were 6 animals per group (3 in pilot studies), randomly assigned based on web based random number generator. Sample size was determined based on expected differences in microvascular plasma flow. Criteria were in place to determine when animals should be euthanized; none were euthanized or died outside of the experimental protocol. Animal health and behavior were monitored twice daily.

*Intravital multi-photon fluorescence microscopy [1, 4, 5].* Microvascular flow was quantified by intravital imaging 24 and 48 hours postischemia using an Olympus Fluoview 1000 confocal/multiphoton microscope, Olympus Corporation, Center Valley PA. Briefly, after ensuring adequate anesthesia with thiobarbital (30mg/kg), organs to be imaged were exteriorized and Hoechst (500ug in 0.5ml physiological saline,

Molecular Probes, Eugene, OR) was injected intravenously to allow visualization of nuclei. Microvascular plasma flow was visualized after infusion of fluorescein conjugated albumin (Molecular Probes, 2mg in 0.5ml physiological saline) and serial images acquired approximately every 0.5 second. Plasma flow was quantified as movement of dye-excluding erythrocytes in line scans (with correction for microvascular angle). Rouleaux were identified as shadows of "stacked coins" moving together in blinded, serial intravital images. Intravascular leukocytes were identified as nucleated (stained with Hoechst) cells within the vasculature. The white blood cells (WBC) were classified as adherent if not free flowing or rolling but stationary (on the capillary wall) for at least 10 seconds. All quantification was performed without knowledge of experimental group.

*Renal gene expression [6, 7]:* quantitative real-time polymerase chain reaction (qRT-PCR) was used to quantify mRNA from rat kidneys. RNA was extracted from kidneys with the RNeasy Plus mini Kit (Qiagen, Germantown MD). AffinityScript cDNA Synthesis Kit (Agilent Technologies, Santa Clara CA) was used to make cDNA from 1 ug mRNA. RNA was subsequently amplified and quantified via Mx3005P QPCR System (Agilent Technologies) along with PrimeTime Gene Expression Master Mix and PrimeTime qPCR Primer Assays (Integrated DNA Technologies, Coralville, Iowa) specific to the rat C3 complement component and intercellular adhesion molecule (ICAM).

*Myeloperoxidase(MPO) Activity [3]:* MPO activity, used as an indicator of neutrophil infiltration, was measured in coded tissue homogenates as previously described[3], using *o*-dianisidine dihydrochloride and $H_20_2$, normalized to protein content and expressed as the percent of the level after sham surgery.

*Statistics.* Data are expressed as means ± 1 standard error. Analysis of variance was used to determine if differences among mean values reached statistical significance. Tukey's test was used to correct for multiple comparisons. Fishers' exact test was used for categorical data. The null hypothesis was rejected at $p<0.05$. Statistics and data analysis were performed in a blinded fashion, without knowledge of experimental group.

REFERENCES (Methods)

1. Dunn KW, Sandoval RM, Kelly KJ, Dagher PC, Tanner GA, Atkinson SJ, et al. Functional studies of the kidney of living animals using multicolor two-photon microscopy. Am J Physiol Cell Physiol. 2002;283(3):C905-16. Epub 2002/08/15. doi: 10.1152/ajpcell.00159.2002. PubMed PMID: 12176747.

2. Kelly KJ. Distant effects of experimental renal ischemia/reperfusion injury. J Am Soc Nephrol. 2003;14(6):1549-58. Epub 2003/05/23. doi: 10.1097/01.asn.0000064946.94590.46. PubMed PMID: 12761255.

3. Dominguez JH, Xie D, Kelly KJ. Cardiac effects of renal ischemia. Am J Physiol Renal Physiol. 2023;324(1):F64-F74. Epub 2022/11/18. doi: 10.1152/ajprenal.00183.2022. PubMed PMID: 36395386.

4. Dominguez JH, Xie D, Dominguez JM, 2nd, Kelly KJ. Role of coagulation in persistent renal ischemia following reperfusion in an animal model. Am J Physiol Renal Physiol. 2022;323:F590-F601. Epub 2022/08/26. doi: 10.1152/ajprenal.00162.2022. PubMed PMID: 36007891.

5. Kelly KJ, Dominguez JH. Treatment of the post-ischaemic inflammatory syndrome of diabetic nephropathy. Nephrol Dial Transplant. 2010;25(10):3204-12. Epub 2010/05/15. doi: 10.1093/ndt/gfq217. PubMed PMID: 20466672.

6. Dominguez JH, Liu Y, Gao H, Dominguez JM, 2nd, Xie D, Kelly KJ. Renal Tubular Cell-Derived Extracellular Vesicles Accelerate the Recovery of Established Renal Ischemia Reperfusion Injury. J Am Soc Nephrol. 2017;28(12):3533-44. Epub 2017/07/28. doi: 10.1681/ASN.2016121278. PubMed PMID: 28747315; PubMed Central PMCID: PMC5698065.

7. Dominguez JM, 2nd, Dominguez JH, Xie D, Kelly KJ. Human extracellular microvesicles from renal tubules reverse kidney ischemia-reperfusion injury in rats. PLoS One. 2018;13(8):e0202550. Epub 2018/08/28. doi: 10.1371/journal.pone.0202550. PubMed PMID: 30148844; PubMed Central PMCID: PMC6110463.

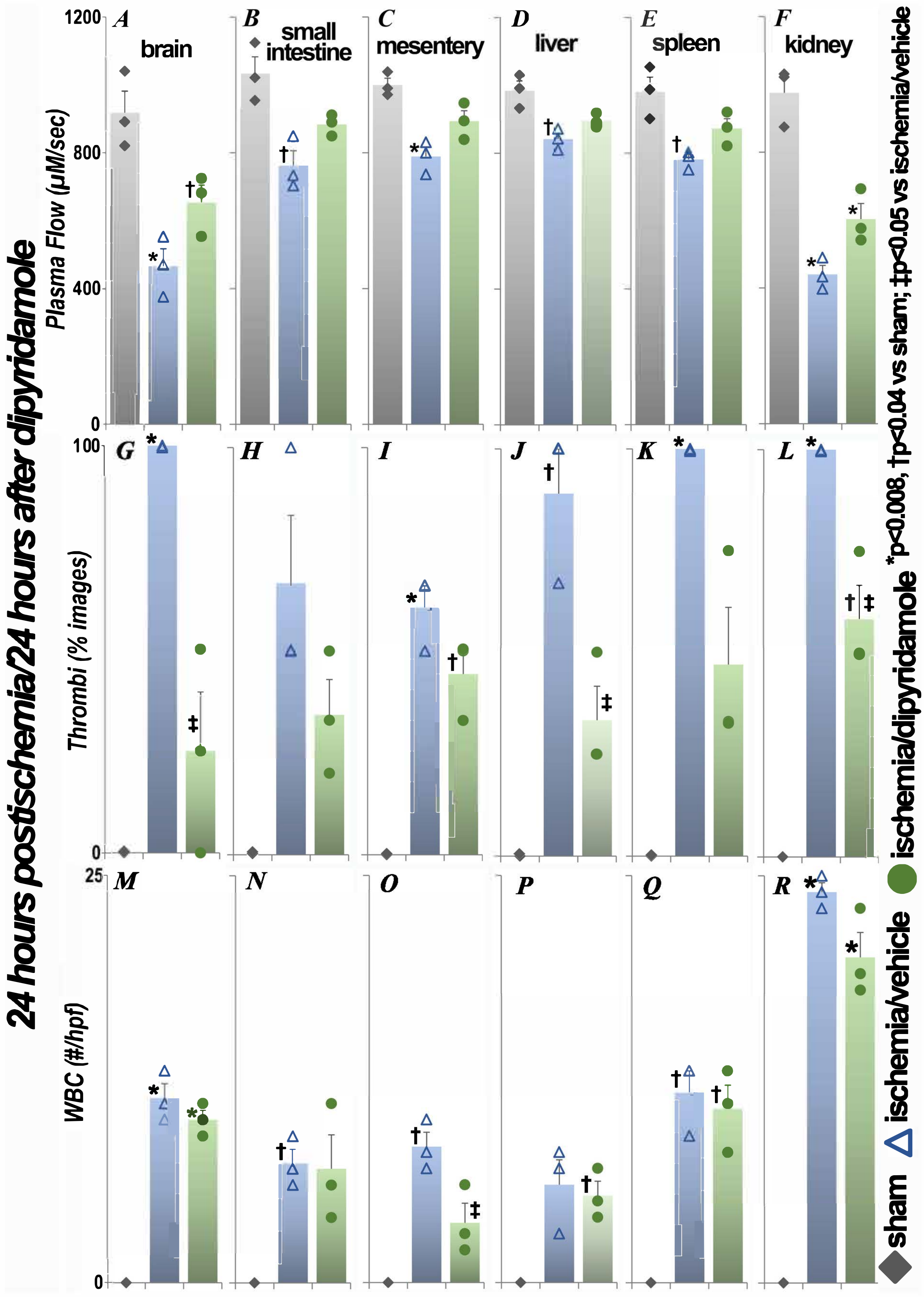
24 hours postischemia/24 hours after dipyridamole
Plasma Flow (μM/sec)
Thrombi (% images)
WBC (#/hpf)
A brain
B small intestine
C mesentery
D liver
E spleen
F kidney
G
H
I
J
K
L
M
N
O
P
Q
R
1200
800
400
0
100
0
25
0
sham
ischemia/vehicle
ischemia/dipyridamole
*p<0.008, †p<0.04 vs sham; ‡p<0.05 vs ischemia/vehicle

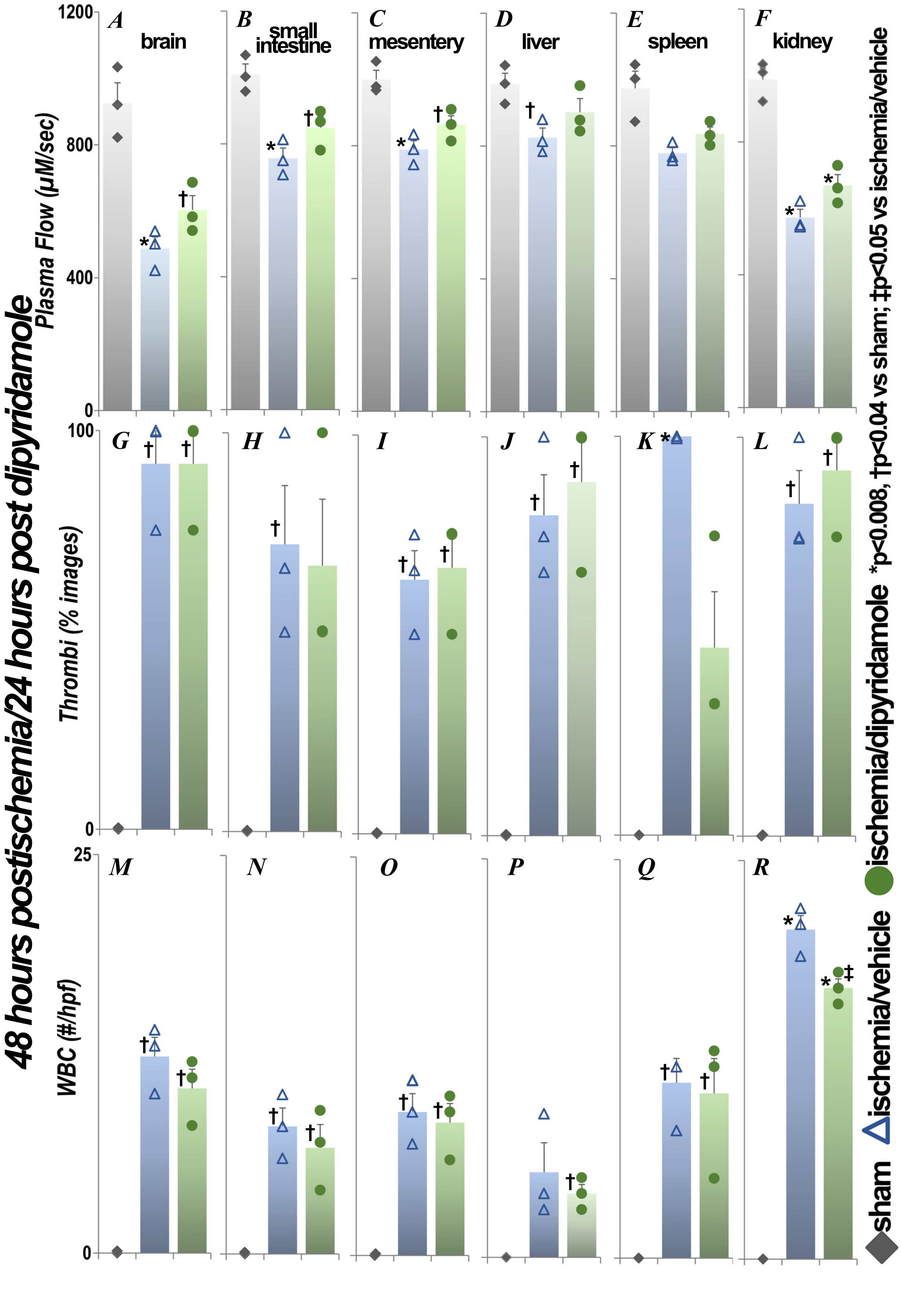
48 hours postischemia/24 hours post dipyridamole
sham
ischemia/vehicle
ischemia/dipyridamole
*p<0.008, †p<0.04 vs sham; ‡p<0.05 vs ischemia/vehicle
Plasma Flow (μM/sec)
Thrombi (% images)
WBC (#/hpf)
A brain
B small intestine
C mesentery
D liver
E spleen
F kidney
G
H
I
J
K
L
M
N
O
P
Q
R
1200
800
400
0
100
0
25
0

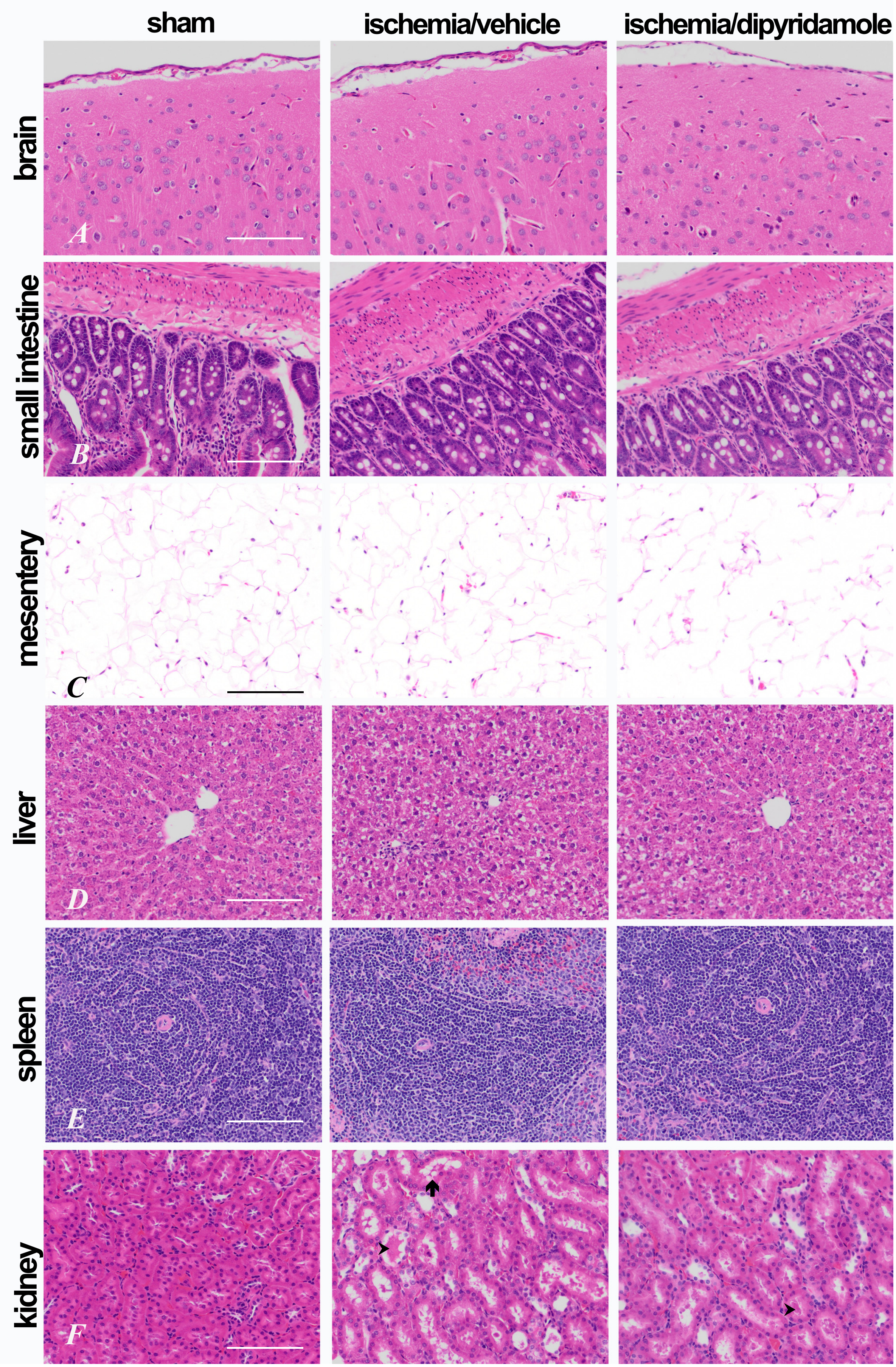
sham
ischemia/vehicle
ischemia/dipyridamole
brain
small intestine
mesentery
liver
spleen
kidney
A
B
C
D
E
F

SUPPLEMENTAL TABLES

TABLE S1

*P VALUES*

| | | Ischemia/vehicle vs sham | Ischemia/dipyridamole vs sham | Ischemia/dipyridamole vs ischemia/vehicle |
|---|---|---|---|---|
| Figure 1 | | | | |
| | RBC aggregation | 9.202E-36 | 3.405E-33 | NS |
| | fibrinogen | 7.277E-07 | 3.4814E-07 | NS |
| | hematocrit | NS | NS | NS |
| | Systolic blood pressure | NS | NS | NS |
| | Serum creatinine | NS | NS | NS |
| | | | | |
| Figure 2 (brain) | | | | |
| | Plasma flow | 0.00252 | NS | 0.0484 |
| | thrombi | 4.01952E-12 | 0.035667663 | 3.3E-09 |
| | WBC | 0.0009379 | 2.30198E-05 | NS |
| | MPO | 0.000925772 | 0.011996 | 0.052163 |
| | C3 | 0.089953 | NS | NS |
| | ICAM | 0.053252 | NS | NS |
| | | | | |
| Figure 3 (small intestine) | | | | |
| | Plasma flow | 0.0024 | 0.02601 | 0.0455 |
| | thrombi | 1.71E-05 | NS | 3.75E-09 |
| | WBC | 0.01293 | 0.015621 | 0.063591 (NS) |
| | MPO | 4.77073E-05 | NS | 3.46E-05 |
| | C3 | 0.046558 | NS | NS |
| | ICAM | NS | NS | 0.07791 |
| | | | | |
| Figure 4 (mesentery) | | | | |
| | Plasma flow | 0.001660 | NS | 0.04947 |
| | thrombi | 6.86717E-06 | 0.001433711 | 0.052376 (NS) |
| | WBC | 0.000724 | 0.080135 (NS) | 0.029808 |
| | MPO | 0.032948872 | NS | 0.048211 |
| | C3 | 0.017689 | NS | 0.044055 |
| | ICAM | 0.036661 | NS | 0.022951 |
| | | | | |
| Figure 5 (liver) | | | | |
| | Plasma flow | 0.04886 | NS | 0.08221 (NS) |
| | thrombi | 2.78971E-12 | 0.03671 | 9.75E-09 |
| | WBC | 0.003396 | 0.02337 | NS |
| | MPO | 0.000125687 | 0.004242 | 0.004908 |
| | C3 | 0.000345 | 0.062736 | 0.068664 |
| | ICAM | 0.049231 | NS | 0.093873 |

| | | | | |
|---|---|---|---|---|
| | | | | |
| Figure 6 (spleen) | | | | |
| | Plasma flow | 0.01494 | NS | 0.001610 |
| | thrombi | 2.5803E-8 | 0.002610 | 0.0009520 |
| | WBC | 0.001489 | 0.024119 | 0.03123 |
| | MPO | 0.011077086 | 0.007004 | 0.063024 |
| | C3 | 0.004803 | 0.045857 | 0.077764 |
| | ICAM | 0.000443 | 0.014384 | 0.045943 |
| | | | | |
| Figure 7 (kidney) | | | | |
| | Plasma flow | 2.100E-5 | 0.000221 | 0.04549 |
| | thrombi | 5.14674E-13 | 0.040727245 | 7.67E-5 |
| | WBC | 0.036108 | 0.019001 | NS |
| | MPO | 0.000225714 | 0.000161 | 0.0041 |
| | C3 | 0.00125 | 0.027472 | 0.015318 |
| | ICAM | 0.002305 | 0.002824 | 0.008488 |
| | | | | |
| Figure 8 (heart) | | | | |
| | esterase | 0.000148 | 0.001936 | NS |
| | MPO | 5.83243E-07 | 0.000282 | NS |
| | C3 | 0.001858 | 0.00026 | NS |
| | ICAM | 0.000296 | 0.0052 | NS |
| | | | | |
| Figure S1 | | | | |
| | Plasma flow--brain | 0.006222 | 0.035207 | 0.061581 |
| | Plasma flow—small intestine | 0.015454 | 0.079956 | 0.09649 |
| | Plasma flow—mesentery | 0.004955 | 0.053279 | 0.067692 |
| | Plasma flow—liver | 0.018178 | 0.071364 | 0.073255 |
| | Plasma flow—spleen | 0.034514 | NS | 0.067196 |
| | Plasma flow—kidney | 0.002452 | 0.005632 | 0.048783 |
| | Thrombi--brain | 1.11E-21 | NS | 0.035099 |
| | Thrombi—small intestine | 0.057191 | 0.058447 | NS |
| | Thrombi--mesentery | 0.0076 | 0.015948 | NS |
| | Thrombi--liver | 0.014935 | 0.057191 | 0.018043 |
| | Thrombi--spleen | 1.11E-15 | 0.078431 | 0.06323 |
| | Thrombi--kidney | 1.11E-13 | 0.019804 | 0.03775 |
| | WBC----brain | 0.006001 | 0.003317 | NS |
| | WBC—small intestine | 0.014156 | 0.078202 | NS |
| | WBC--mesentery | 0.011015 | 0.092735 | 0.039568 |

| | | | | |
|---|---|---|---|---|
| | WBC--liver | 0.059125 | 0.026271 | NS |
| | WBC--spleen | 0.012811 | 0.018054 | NS |
| | WBC--kidney | 0.000578 | 0.005783 | NS |
| | | | | |
| Figure S2 | | | | |
| | Plasma flow--brain | 0.007139 | 0.015993 | NS |
| | Plasma flow—small intestine | 0.00465 | 0.027767 | NS |
| | Plasma flow—mesentery | 0.005135 | 0.023909 | NS |
| | Plasma flow—liver | 0.022924 | NS | NS |
| | Plasma flow—spleen | 0.052037 | NS | 0.099581 |
| | Plasma flow—kidney | 0.000886 | 0.002496 | 0.083956 |
| | Thrombi--brain | 0.008163 | 0.008163 | NS |
| | Thrombi—small intestine | 0.039459 | 0.057191 | NS |
| | Thrombi--mesentery | 0.01293 | 0.015268 | NS |
| | Thrombi--liver | 0.015654 | 0.015948 | NS |
| | Thrombi--spleen | 1.11E-07 | 0.078529 | 0.06323 |
| | Thrombi--kidney | 0.009852 | 0.008163 | NS |
| | WBC----brain | 0.009363 | 0.013259 | NS |
| | WBC—small intestine | 0.020204 | 0.044363 | NS |
| | WBC--mesentery | 0.016065 | 0.020173 | NS |
| | WBC--liver | NS | 0.020204 | NS |
| | WBC--spleen | 0.018743 | 0.04195 | NS |
| | WBC--kidney | 0.001816 | 0.001151 | 0.032199 |

TABLE S2

*THROMBI—48 hours post ischemia/48 hours after dipyridamole*

| | | SHAM | ISCHEMIA/VEHICLE | ISCHEMIA/DIPYRIDAMOLE |
|---|---|---|---|---|
| BRAIN | numerator | 1 | 28 | 8 |
| | denominator | 23 | 28 | 32 |
| SMALL INTESTINE | numerator | 0 | 22 | 4 |
| | denominator | 16 | 34 | 28 |
| MESENTARY | numerator | 0 | 26 | 12 |
| | denominator | 16 | 39 | 28 |
| LIVER | numerator | 0 | 47 | 4 |
| | denominator | 24 | 56 | 24 |
| SPLEEN | numerator | 0 | 11 | 6 |
| | denominator | 20 | 11 | 16 |
| KIDNEY | numerator | 0 | 64 | 5 |
| | denominator | 16 | 72 | 12 |

*THROMBI—24 hours post ischemia/24 hours after dipyridamole*

| | | SHAM | ISCHEMIA/VEHICLE | ISCHEMIA/DIPYRIDAMOLE |
|---|---|---|---|---|
| BRAIN | numerator | 0 | 17 | 4 |
| | denominator | 16 | 17 | 14 |
| SMALL INTESTINE | numerator | 0 | 13 | 5 |
| | denominator | 17 | 20 | 14 |
| MESENTARY | numerator | 0 | 11 | 9 |
| | denominator | 16 | 18 | 20 |
| LIVER | numerator | 0 | 15 | 5 |
| | denominator | 16 | 17 | 14 |
| SPLEEN | numerator | 0 | 17 | 9 |
| | denominator | 17 | 17 | 17 |
| KIDNEY | numerator | 0 | 19 | 10 |
| | denominator | 17 | 19 | 18 |

*THROMBI—48 hours post ischemia/24 hours after dipyridamole*

| | | SHAM | ISCHEMIA/VEHICLE | ISCHEMIA/DIPYRIDAMOLE |
|---|---|---|---|---|
| BRAIN | numerator | 0 | 14 | 15 |
| | denominator | 16 | 15 | 16 |
| SMALL INTESTINE | numerator | 0 | 13 | 11 |
| | denominator | 17 | 18 | 17 |
| MESENTARY | numerator | 0 | 10 | 9 |
| | denominator | 17 | 16 | 14 |
| LIVER | numerator | 0 | 12 | 15 |
| | denominator | 18 | 15 | 17 |
| SPLEEN | numerator | 0 | 18 | 9 |
| | denominator | 17 | 18 | 17 |
| KIDNEY | numerator | 0 | 15 | 15 |
| | denominator | 15 | 18 | 16 |
